# Emphysema after Sinus Grafting: Importance of Patient's Information, Early Diagnosis, and Management

**DOI:** 10.1155/2020/1525673

**Published:** 2020-07-28

**Authors:** Marc El Hage, Nathalie Nurdin, Mark Bischof, Rabah Nedir

**Affiliations:** ^1^Ardentis Clinique Dentaire Lausanne, Swiss Dental Clinics Group, Voie du Chariot 6, 1003 Lausanne, Switzerland; ^2^Ardentis Clinique Dentaire Vevey, Swiss Dental Clinics Group, Rue du Collège 3, 1800 Vevey, Switzerland

## Abstract

The sinus elevation procedure is a safe and predictable technique that allows the placement of implants in atrophic posterior maxillae. However, some recommendations have to be followed by the patient to ensure reliable healing. It is particularly important to avoid inducing trauma in the region concerned and through the sinuses. This report describes a rare complication that occurred after the grafting of a sinus, which was attributed to a violent sneeze a few hours after the intervention. The diagnosis of emphysema following air entry was confirmed by the suddenness of the swelling and associated crepitation, and by the radiographic observation of a delimited radiolucent zone in the grafted sinus. The immediate diagnosis and subsequent management prevented further adverse events. This case report supports the need for complete comprehensive instruction of patients after oral surgery, swift diagnosis, and management of emphysema.

## 1. Introduction

Sinus grafting is often necessary when the residual bone height under the sinus is not sufficient to rehabilitate maxillary sites with implant-supported prostheses. Implants can be placed simultaneously with grafting or during additional surgery after allowing for a healing period of the grafted region. After each surgical procedure, patients are informed of possible complications. The parameters that may compromise the healing are also described, such as smoking, poor oral hygiene, and behaviors that may cause trauma to the surgical site.

Emphysema induced by the introduction of air through tissues during a dental procedure is not common. It can be associated with the following treatments: crown removal and subgingival curettage, tooth extraction, orthodontic miniscrew placement, class-V resin fillings, tooth preparation, and root canal treatment [1]. The use of high-speed handpieces and air/water syringes can induce this complication [2]. The entry of air through the tissues generally results in facial or cervico-facial swelling that appears immediately after the dental intervention. Crepitation may accompany clinical palpation of the swelling region due to the air entrapped in the tissues. Emphysema can extend to the neck and thorax, and lead to severe complications when a large amount of air reaches the mediastinum and the pleural space [2, 3].

This report describes a case of emphysema that occurred after the grafting of a sinus as the first step of implant treatment to rehabilitate a missing single tooth in the maxillary molar region. The complication was related to the forced entry of air via the maxillary sinus after a violent sneeze. The immediate diagnosis of emphysema and subsequent management prevented further adverse events.

## 2. Case Presentation

### 2.1. Initial Patient Assessment

**Figure 1:**
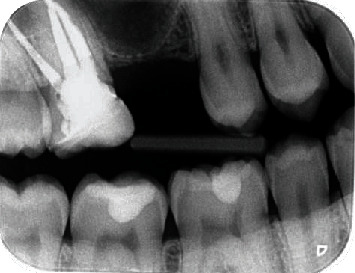
Bitewing radiography. Initial situation.

In November 2013, a 28-year-old Caucasian man visited the office for the replacement of the upper right first molar, which had been lost some years previously. The patient presented good oral hygiene and was not a smoker. During the clinical examination, the patient did not report any pain in the region of the maxillary sinuses. Bitewing radiography showed an extremely low residual bone height at the site of the upper right first molar. The upper right second molar was subject to endodontic root canal treatment (Figure 1). The general medical and dental history did not reveal any particular problem or symptoms related to maxillary sinusitis.

**Figure 2:**
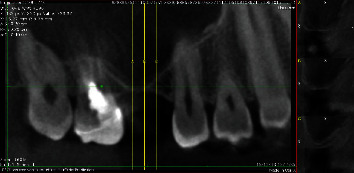
CBCT. Initial situation.

After discussion, the patient chose to receive an implant with a single crown rather than a fixed prosthesis of three units supported by the maxillary right second premolar and molar. Cone-beam computerized tomography (CBCT; Model CS 9300, Carestream Health, Inc., Rochester, New-York, USA) performed before surgery did not demonstrate the presence of a septum at the former position of the upper right first molar. The residual bone height was 1.0 mm (Figure 2).

### 2.2. Sinus Grafting

**Figure 3:**
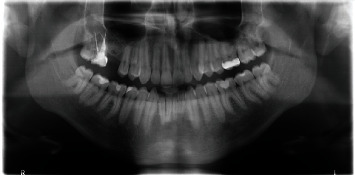
Panoramic radiograph taken immediately after sinus grafting.

Lateral sinus floor augmentation with a xenograft was conducted. Antibiotic prophylaxis was started within 1 hour before the surgery (Dalacin® C, Pfizer SA, Zürich, Switzerland, 300 mg) and was administered for 5 days (300 mg, 3 times per day). Local anaesthetic (Ubistesin™, 3 M ESPE AG, Germany) was infiltrated at the vestibular and palatal areas of the sites of the upper right second premolar to the second molar. A mucoperiosteal access flap was created with a crestal incision and two vertical incisions in order to expose the lateral wall of the maxillary sinus. A bony lateral window was obtained with a piezoelectric device. To decrease the risk of perforation, the Schneiderian membrane was carefully elevated using flat-head ultrasonic inserts. After its complete elevation, the membrane was reflected to obtain a space large enough for the grafting material. No perforation of the membrane was clinically observable immediately after its elevation. The integrity of the membrane was controlled by using the Valsalva maneuver. A mixture of deproteinized bovine bone material (Bio-Oss®, Geistlich AG, Wolhusen, Switzerland) and the patient's blood collected at the surgical site were then inserted. The lateral window was covered with two layers of a resorbable porcine collagen membrane (Bio-Gide®, Geistlich Pharma AG, Switzerland). The flap was closed without tension using nonresorbable polyamide sutures (Suturamid® 4.0, B. Braun Aesculap, Sempach, Switzerland). Postoperative examination was carried out with a panoramic radiograph (Figure 3), which validated the insertion of the graft in the space created under the nonperforated membrane. Standard postoperative recommendations were made, including not blowing the nose during the healing period and avoiding traumatic behaviors that may have deleterious effects on the treated region.

**Figure 4:**
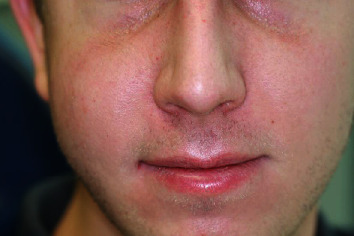
Photograph taken one day after the sinus grafting showing the unilateral tumefaction in the right genian region.

**Figure 5:**
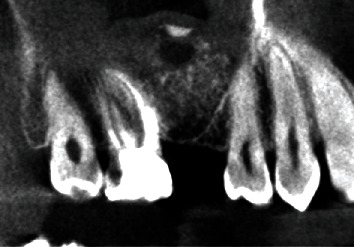
CBCT obtained one day after the sinus grafting. A delimited radiolucent zone can be observed in the grafted region.

One day after surgery, the patient came to the clinic and requested an examination. A few hours after the procedure, he had sneezed violently while keeping his mouth closed. He had immediately felt the swelling of the genian region with a simultaneous sound of cracking. The clinical examination confirmed unilateral tumefaction of the right genian region accompanied with crepitation on palpation (Figure 4). CBCT was performed in order to check the integrity of the Schneiderian membrane and the stability of the grafting material under the sinus, without spreading inside the sinus. However, it did reveal a delimited radiolucent zone inside the grafted region (Figure 5). The diagnosis of emphysema by air entry was thus made. Treatment with metronidazole (Flagyl® 500 mg, Sanofi-Aventis SA, Vernier, Switzerland, 500 mg, 3 times per day for 7 days) was then introduced in addition to the existing antibiotic medication. Examinations were made two and ten days afterwards (at suture removal). The grafted site healed completely without any symptoms of further complications.

### 2.3. Implant Placement

**Figure 6:**
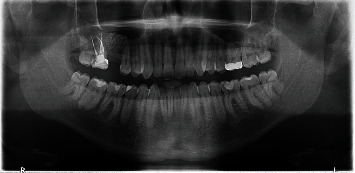
Panoramic radiograph. Preoperative situation before implant placement.

Ten months after sinus grafting, the implant was placed. First, preoperative panoramic radiography was performed to examine the graft (Figure 6). A radiographic bone height of 14 mm was measured at the site to be implanted.

**Figure 7:**
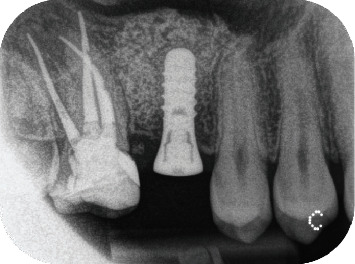
Periapical radiograph obtained immediately after implant placement.

The implant bed was prepared according to the manufacturer's instructions, followed by the insertion of a Standard Plus Regular Neck implant with a SLActive® surface (diameter: 4.1 mm, length: 10 mm; Straumann AG, Basel, Switzerland). A periapical radiograph was obtained immediately after implant placement (Figure 7). The sutures were removed after 10 days. Healing was uneventful.

### 2.4. Prosthetic Restauration and Follow-Up

**Figure 8:**
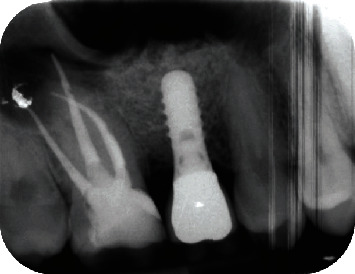
Periapical radiograph obtained one year after implant placement.

Ten weeks after surgery, the abutment was tightened with a torque of 15 Ncm. A ceramo-metal crown was cemented and functionally loaded. The clinical and radiographic examination after one year showed a stable situation and the implant in function (Figure 8).

## 3. Discussion

In oral implantology, the standard clinical practice used to treat the presented case required a sinus augmentation procedure with graft insertion and delayed implant placement [4]. With such an atrophic site, a lateral approach with the use of grafting material and delayed implant placement was recommended [5]. The main intraoperative concerns with the lateral window approach are perforation of the Schneiderian membrane, graft migration, haemorrhagic accident, and postoperative acute or chronic sinus infection, bleeding, wound dehiscence, exposure of the barrier membrane, and graft loss [6, 7].

Prevalence of damages to the Schneiderian membrane during a sinus elevation and grafting was estimated about 10 to 50%. Most damages are 1- to 3-mm tears and regenerate spontaneously. When tears and lacerations are detected, the sinus membrane can be protected with biological resorbable membranes [7–9]. Such complications may compromise the outcomes of the inserted graft by resulting contamination and infection. The sinus graft infection is considered a major complication requiring urgent treatment, based on the risk of infection which could spread throughout the graft and sinus cavity or the adjacent anatomical structures. It is rare between 1 and 4% [8] and occurred more frequently in patients with a history of sinusitis [10]. Various treatments were proposed such as systemic antibiotics, endoscopy of the sinus, surgical exploration, rinsing, and/or curettage of the affected maxillary sinuses to totally remove inserted biomaterials and inflammatory tissue [11].

In the present article, no perforation of the membrane was clinically observable immediately after its elevation. The integrity of the membrane was controlled by using the Valsalva maneuver.

Introduction of air and consequent emphysema are known and described in the literature. Emphysema is considered an early adverse event, and this acute complication needs emergency treatment [1]. This report presents a rare and minor case of emphysema after sinus grafting due to a strong sneeze with a closed mouth. The positive pressure created by the sneeze led to the spread of air through the soft tissues and consequent facial swelling. Keeping the mouth closed increased the internal antral pressure. The medical history of the patient was noncontributory, without sinus-related problems. The CBCT taken one-day later has not shown opacity of the sinus and thickening of the Schneiderian membrane, usually observable when the sinus is affected. No oroantral communication was detectable. Therefore, the risk of graft infection was discarded. Emphysema was then quickly diagnosed, which was confirmed by the presence of a delimited radiolucent zone in the grafted region and the crepitation on palpation. The swelling was localized. Therefore, treatment covering specifically anaerobic microorganisms in combination with the initial antibiotic prescription was administered. This treatment and close monitoring of the patient were sufficient to resolve the complication. The event did not compromise the placement of the implant. The implant procedure and management of its complication have led to a successful outcome over one year of follow-up.

Emphysema is more common after dental procedures involving the maxilla or posterior region than after those involving the mandible or anterior region [1]. In the majority of cases of subcutaneous emphysema, a diffuse swelling is observable. A conservative management is carried out, and an antibiotic therapy is indicated in order to prevent soft tissue abscessation. Spontaneous resolution is then expected; the emphysema is likely to decrease within 4 to 7 days [12].

The complete history of the patient is of primary importance to the diagnosis. A rapid swelling may be also *inter alia* the sign of allergic reaction. Clinical palpation is the first action required to evaluate the area concerned. Crepitation and/or an air bubble consistency under finger pressure is often felt by the patient. Symptoms of fever and nausea may indicate infection. A history of periodontal disease may be a contributory factor for further severe complications [13]. The use of CBCT is needed to detect and evaluate the spread of air. Rapid diagnosis and strict follow-up of the patient avoid further potentially fatal complications such as venous air embolism and/or soft tissue infections [2, 3, 14, 15].

Most cases of emphysema caused by intrusion of air after dental surgery are iatrogenic, for example, due to the use of rotary surgical tools or air syringes during the procedure [16]. Cases due to the postoperative behavior of the patient following maxillary sinus floor grafting are rare. Sakakibara et al., Farina et al., and Sevilla Heras et al. described sinus elevation and sudden facial swelling that appeared after the patients blew their nose roughly or sneezed [17–19]. These cases were not severe, although the swelling extended to the peri-orbital and orbital regions [18, 19].

## 4. Conclusions

Subcutaneous emphysema resulting from the entry of air can occur as a complication of a sinus grafting procedure in an atrophic posterior maxilla. The causes for such a complication include sneezing while the mouth closed. This case report supports the need to provide complete comprehensive instructions to patients after oral surgery and more specifically sinus grafting. It also indicates the importance of an immediate diagnosis. The swift management of emphysema is vital to avoid potentially serious adverse events. It should include the use of CT to evaluate the location and importance of air-filled spaces.
